# Pharyngeal hairy polyps: Case series and literature review

**DOI:** 10.1097/MD.0000000000014305

**Published:** 2019-02-01

**Authors:** Yishu Teng, Zhixiong Xian, Saihong Han, Zhenjiang Liang, Hongguang Pan, Lan Li

**Affiliations:** Department of Otolaryngology, Shenzhen Children's Hospital, Shenzhen, Guangdong, China.

**Keywords:** child, hairy polyp, pathology, pharynx, teratoma

## Abstract

It was aimed to report 5 cases of pharyngeal hairy polyps, and to summarize the characteristics combined with literature review.

Five cases with pathological diagnosis of pharyngeal hair polyps were diagnosed and treated in our department from June, 2006 to October, 2016, and retrospective analysis of their clinical data was performed. Among the 5 cases, there were 1 male and 4 female, with the age of 2 days to 26 months old. After birth, these patients were accompanied by stridor, difficulty breathing, snoring, feeding difficulties, and slow weight gain.

Gray mass in the stem original from the pharynx was found in all 5 cases, with the surface hair-covering. The polyp resections were performed under general anesthesia, with the complete removal of polyp along the pars basilaris during surgery smoothly. The operation during was 5 to 20 minute, with an average of 12 minute, and there was little hemorrhage during operation. Symptoms disappeared completely after the surgery, and follow-up was performed for 1 year without recurrence shown.

Pharyngeal hairy polyp is a rare non-malignant clinical disease, mainly caused by symptoms in respiratory tract obstruction. Complete removal of polyps along the pars basilaris is an effective treatment, with no recurrence case reported after surgery.

## 1 Introduction

Pharyngeal hairy polyp is a rare developmental abnormality of epithelium, which contains ectoderm and mesoderm. In clinic, the manifestations are as polyps, with the skin-like tissues and covered with hair and sebaceous glands. Hairy polyps occur in newborns, infants and young children mostly, and the incidence of female is greater than male (with the ration of male to female of 1: 6), without unknown origin.^[1]^ Most of hairy polyps occur in the nasopharynx or pharynx oralis (with the most part on the left side), while a small part occurs in the soft palate, hard palate, tongue, tonsil or tympanum.^[2,3]^ The patients often accompanied by stridor, difficulty breathing, snoring, feeding difficulties, and slower weight gain than children at the same age. Missed diagnosis and misdiagnosis often occur, and surgical resection is an effective way to treat this disease. A total of 5 cases of pharyngeal hairy polyps diagnosed and treated in Department of Otolaryngology, Shenzhen Children's Hospital from June, 2006 to October, 2016 with satisfactory results, are summarized as follows.

## 2 Material and methods

### 2.1 Clinical data

**Figure 1:**
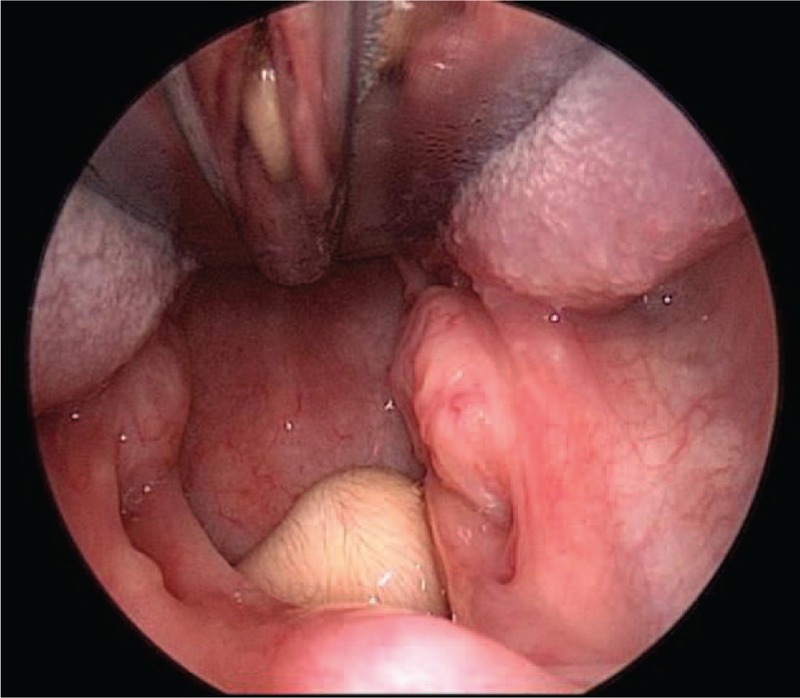
Visible gray mass on the lateral wall of the nasopharynx (patient # 5).

**Figure 2:**
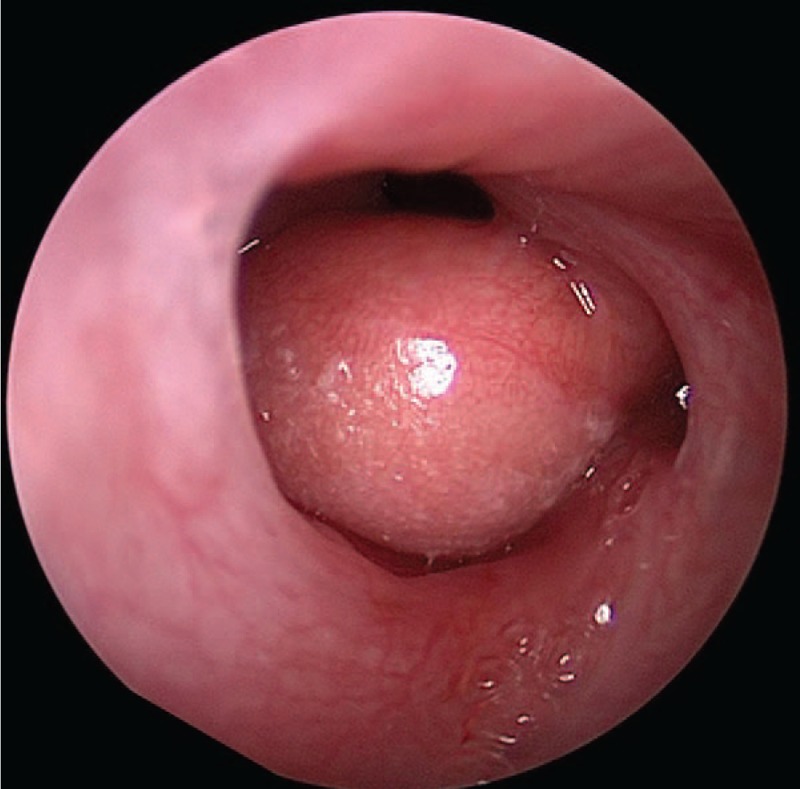
Hairy polyps on the right side of the nasopharynx (patient #1).

**Figure 3:**
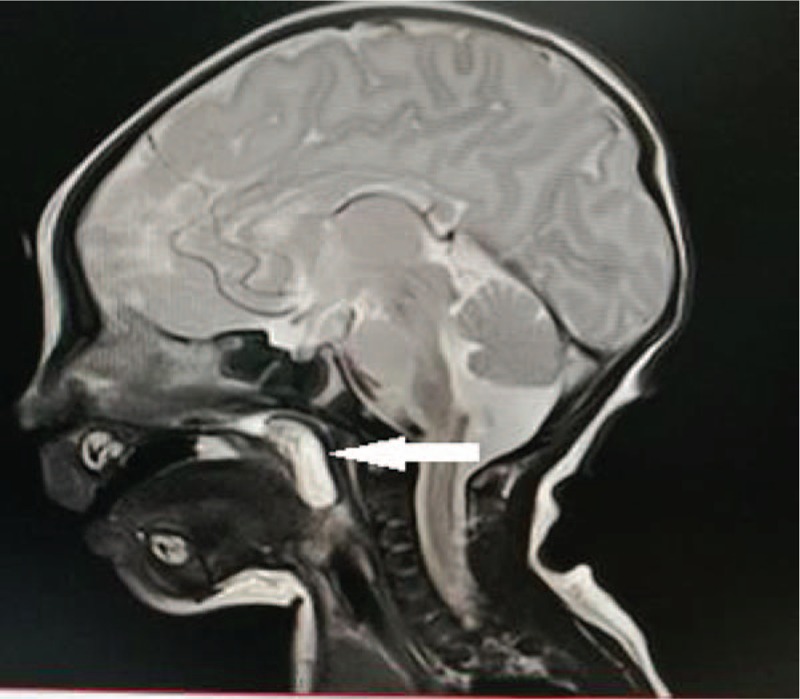
Clear boundaries of T2-weighted MRI image prompted adipose-derived mass (patient #1).

Among the 5 cases in the current study, there were 1 male and 4 female, with the age of 2 days to 26 months old. They were hospitalized with the main complaints of stridor, snoring, and difficulty breathing etc. Clinical data of 5 patients are shown in Table 1. Pharyngeal masses were found in 2 cases immediately after the birth, in 1 case after 3 weeks, in 1 case after 2 months, and in 1 case after 26 months. The original locations of these 5 cases were as follows: The original locations of these 5 cases were as follows: 4 cases from the nasopharynx and 1 case from pharynx, which was from the edge of soft palate. The masses were cylindrical, with less smooth surface and skin-like appearance, without congestion, and with positive activity (as shown in Figs. 1 and 2). Magnetic Resonance Imaging (MRI) examination has prompted the nasopharyngeal mass (as shown in Fig. 3). All of the 5 patients were challenged with varying degrees of airway obstruction, such as stridor, three depression sign and snoring. The publication of cases report is exempted from approval by the Institutional Review Board of Shenzhen Children's Hospital. However, Informed consent was obtained from these patients’ parents for the publication of this cases report.

**Table 1:**
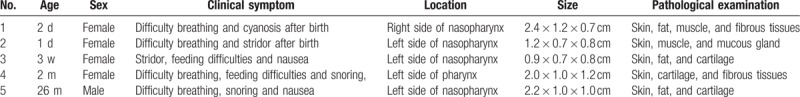
Clinical manifestations of hairy polyps in 5 cases.

### 2.2 Surgical methods

Routine preoperative examinations were performed in all 5 cases. Under general anesthesia in tracheal intubation, pharynx was the exposed with mouth gag. The visible hairy mass is single, with long pedicle and skin-like surface (as shown in Figs. 1 and 2). Endoscope was installed from the nasal cavity; it can be seen that the pars basilaris of 4 cases were on the left side of the back of soft palate and that of 1 case was in the oropharynx. The pars basilaris was fixed with vessel clamp, with a single pole electric knife cutting off, and coagulation performing; there was no bleeding during surgery.

## 3 Results

**Figure 4:**
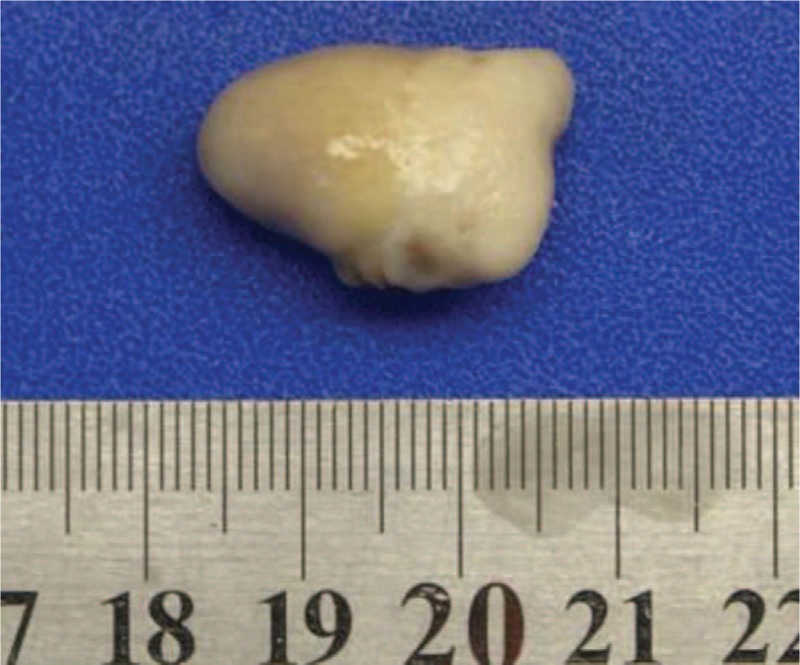
Hairy polyps (patient #5).

**Figure 5:**
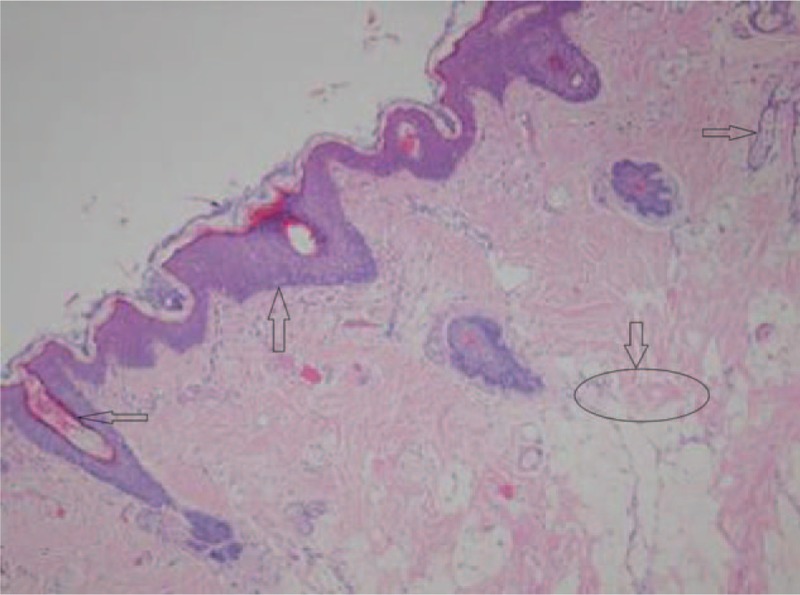
Epidermal keratinized stratified squamous epithelium, with fibrous adipose tissues, hair follicles and sebaceous glands in subcutaneous tissues (patient #1). Epidermal keratinized stratified squamous epithelium 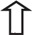 fibrous adipose tissues 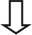 hair follicles 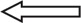 sebaceous glands 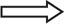.

The 5 patients were successfully operated, with the operation time of 5 to 20 minutes and an average of 12 minutes. There was no intraoperative bleeding; the postoperative masses were sent for pathological examinations. The pathological examination results were as follows: masses were soft with gray color, clear boundary, and substantiality. Microscopic examination results were as follows: the surface of the hairy polyps were like skins made up of the soft keratinocyte layer, hair follicles and sebaceous glands, in which, the core part was fibrous adipose tissues, usually accompanied by focal cartilage, muscle and bone (as shown in Figs. 4 and 5). After surgery, symptoms like respiratory obstruction, snoring and feeding difficulties disappeared. The 5 cases were followed up for more than 1 year, with no recurrence occurred.

## 4 Discussion

Pharyngeal hairy polyp is a rare congenital benign disease, with the incidence rate of 1/40,000 from foreign literature statistics,^[4–6]^ which is a soft pedunculated tongue-like mass, with hairy surface, presenting gray or slightly purple;^[2]^ the clinical manifestation is as polyp. It often occurs in newborns, infants and young children; the incidence of female is higher than that of male (with the ratio of male to female = 1: 6). In the current case report, the ratio of male to female was 1: 4, which is consistent with the above-mentioned incidence. 60% of the pars basilaris of pharyngeal hairy polyps are located mostly in the nasopharyngeal wall or soft palate back, and partly in the hard palate, auris media, eustachian tube, tongue or tonsil. Most cases are located on the left side of nasopharynx or oropharynx, which is consistent with the pathogenesis of 5 children in this study. The polyp occurring in the tonsils has an earlier onset than in other parts. Planas et al reported 1 case occurred in the embryonic period, high-risk pregnancy after the termination of the diagnosis of hairy polyps.^[7]^

Hairy polyp is a manifestation of rare developmental abnormalities in epithelium, which includes ectoderm and mesoderm. Ectodermal development and differentiation form squamous epithelial tissues, with the surface, showed gray. Endodermal development and differentiation contribute into fibrous fat including muscle, cartilage, glandular tissue, fat and a few neuroepithelial tissues. Hairy polyps have been classified as teratomas, dermoid cysts and choristomas. However, there are 3 differences between hairy polyp and teratoma, which are listed as follows: 1. The morbidity of teratoma with the ratio of male to female is quite similar, but that of hairy polyp is 6: 1; 2. Hairy polyp contains mesoderm and ectoderm, but teratoma contains all endoderm, mesoderm and ectoderm ^[6]^; 3. There was no malignant report on hairy polyps, but teratoma can be malignant. Hairy polyp is most likely to belong to teratoma in histopathology, which is benign mass with a normal tissue structure growing in the abnormal anatomical location. It is often considered as congenital malformations or dysplasia,^[6]^ instead of tumors like teratoma or dermoid cyst. This is similar to hairy polyp.

Currently, the pathogenesis of hairy polyps is not clear, but there are several possible opinions. On one hand, choristoma may be derived from the multifunctional tissue, among which, the multifunctional tissue with differentiating potentiality escapes in the form of irregular masses, thus forming a choristoma. They may also be a part of nasopharyngeal mucosa; the nasopharyngeal mucosa does not digest in 7 weeks of pregnancy normally, instead, they live with a long-term existence. On the other hand, another theory is due to malformation in the embryonic formation of the first and the second branchial arch.^[8]^

Soon or shortly after birth, hairy polyps are usually diagnosed, and if the mass is small without symptoms, it may be found until growing up. In the current case report, among the 5 cases of children, 1 case was found due to upper respiratory tract infection until treated in the pediatric department after 2 years old. The clinical symptoms of hairy polyps depend on the size and location, and breathing disorder is the main feature of the disease. If the polyps are small, patients usually suffer from stridor and intermittent breathing difficulties after birth; if the polyps are large, suffocation can be caused.^[9]^ Some other symptoms include vomiting, feeding difficulties, nostrils incitement, cyanosis, recurrent cough, and snoring. Hairy polyp is usually presented as an independent disease, which is often combined with cleft palate, uvula dysplasia, microtia and facial development asymmetry; in addition; there were reports presenting the relationship with the rare hypothalamic neuronal hamartoma and the first branchial cleft fistula on the same side.^[10]^ In this paper, the 2-year-old patient with microtia in left ear, the pars basilaris of the mass in the right side of nasopharyngeal part was combined with the polydactyly of the right thumb; the remaining children showed no significant developmental abnormalities.

Radiology is important for the evaluation of the origin and extent of nasopharyngeal and oropharyngeal lesions in infants and young children, helping to identify the diagnosis and determination of intracranial violation, as well as the surgical program. Computed Tomography examination can assess the mass and its surrounding sclerotin, at the same time, X-ray radiation should be considered. MRI examination can figure out the characteristics and scopes, as well as the relationship with the blood vessels, helping for diagnosis and surgical program development. The dermatoglyphic image of hairy polyps is characterized as polypoid mass composed of fat and fibrous tissues, without intracranial or spinal violations. The high signal in the T1WI image is the expression of adipose tissues in hairy polyps. The high-fat component can narrow nasopharynx of infants and young children. The differential diagnosis of oropharyngeal masses is diagnosed as hamartoma, teratoma and dermoid cysts, which helps to exclude some diseases such as neuroblastoma, hemangioma, and encephalomeningocele, etc.^[11]^

Pharyngeal hairy polyp is a rare benign tumor, with slow growth and no bleeding; there are a few domestic cases reported. Clinical manifestations are mainly presented as airway obstruction caused by a series of symptoms, such as intermittent dyspnea, snoring and feeding difficulties. Complete resection along the pars basilaris is the best treatment, without recurrence shown, and 5 cases in this case report were followed up for more than 1 year without recurrence. Pharyngeal hair polyp is rare in clinic, but the possibility of hairy polyps should be taken into account in dealing with infants suffering from airway obstruction, because the nasopharyngeal hairy polyp is easy to be misdiagnosed. There was 1 case of newborn with dyspnea after birth reported, the symptom of dyspnea had a remission after tracheal intubation, but the patient had delayed treatment considering of laryngeal tracheal disease.^[12]^

## 5 Conclusions

Pharyngeal hairy polyp is a rare non-malignant clinical disease, mainly caused by symptoms in respiratory tract obstruction. Accurate diagnostics must be performed in depth so that proper surgical treatment may be performed.^[13]^ Complete removal of polyps along the pars basilaris is an effective treatment,^[14]^ with no recurrence case reported after surgery.

## Author contributions

**Conceptualization:** Saihong Han, Hongguang Pan, Lan Li.

**Data curation:** Yishu Teng, Zhixiong Xian, Zhenjiang Liang, Hongguang Pan, Lan Li.

**Formal analysis:** Yishu Teng, Zhixiong Xian, Saihong Han, Zhenjiang Liang, Hongguang Pan, Lan Li.

**Funding acquisition:** Yishu Teng, Saihong Han, Zhenjiang Liang.

**Investigation:** Yishu Teng, Zhixiong Xian, Saihong Han, Zhenjiang Liang, Lan Li.

**Methodology:** Yishu Teng, Saihong Han, Zhenjiang Liang, Hongguang Pan, Lan Li.

**Project administration:** Yishu Teng, Hongguang Pan, Lan Li.

**Resources:** Yishu Teng, Zhenjiang Liang, Hongguang Pan, Lan Li.

**Software:** Yishu Teng, Zhixiong Xian, Saihong Han, Lan Li.

**Supervision:** Yishu Teng, Saihong Han, Hongguang Pan.

**Validation:** Yishu Teng, Zhixiong Xian, Hongguang Pan.

**Visualization:** Yishu Teng, Zhixiong Xian, Saihong Han.

**Writing – original draft:** Yishu Teng, Zhixiong Xian, Zhenjiang Liang.

**Writing – review & editing:** Yishu Teng, Zhenjiang Liang.
